# Occult Suicidality and Psychiatric Disease Among Emergency Department Patients with Low-acuity Chief Complaints

**DOI:** 10.5811/westjem.2018.2.36399

**Published:** 2018-03-13

**Authors:** Stephen M. McBride, Valerie A. Braz, Christopher W. Jones

**Affiliations:** Cooper Medical School of Rowan University, Department of Emergency Medicine, Camden, New Jersey

## Abstract

**Introduction:**

Patients presenting to emergency departments (ED) are often screened for suicidality, even when their chief complaint does not involve mental health concerns. Patient receptiveness to ED-based mental health screening and intervention is unknown, particularly among patients with low-acuity chief complaints, who often prioritize rapid evaluation and discharge.

**Methods:**

This cross-sectional study included adults with low-acuity chief complaints presenting to an urban, academic ED in the Northeastern United States during daytime and evening hours, from 2015 to 2016. Participants completed validated mental health screening instruments, including the Suicide Behaviors Questionnaire-Revised and the Patient Health Questionnaire-4. Participants were also asked to rate the importance of addressing mental health concerns during their ED visit.

**Results:**

We approached 1,688 patients, and 816 (48.4%) consented to participate in the study. Of these, 27% screened positive for anxiety and 25% screened positive for depression. Even among patients with no prior depression history, 17% were at high risk of depression. Eleven percent of participants were at high risk for suicidal behavior, including 5% of those with no reported history of depression or bipolar disorder. Thirty-five percent of patients at risk for suicide and 53% of those at high risk of depression thought it was important or very important to address these issues during the ED visit.

**Conclusion:**

Symptoms of mental health disorders were common among this group of ED patients presenting with low-acuity chief complaints. Patients often desired to address these mental health concerns as part of their ED visit.

## INTRODUCTION

Suicide is a leading cause of death in the United States, particularly among younger adults.^1^–^3^ Each year U.S. emergency departments (ED) treat approximately half a million patients for attempted self-harm or suicide.^4^ Prior studies have demonstrated that patients seeking ED care for issues not related to mental health have significant rates of depression and occult suicidal thoughts.^5^,^6^ As a result, the Joint Commission has mandated that U.S. EDs screen all patients for suicidal ideation.^7^ Despite this mandate, little is known about the effectiveness of broadly implemented mental health screening programs, and the U.S. Preventive Services Task Force has concluded that insufficient evidence exists to support generalized screening for suicide risk.^8^ Importantly, the link between screening for mental health disorders and improved patient outcomes depends in part on the receptiveness of patients to interventions that might be implemented when screening suggests the presence of a disorder such as suicidal ideation.

Patients presenting to the ED with low-acuity chief complaints comprise up to two-thirds of ED visits, and may face unique barriers to mental health screening.^9^ For example, because these patients are often rapidly treated and discharged, and because they often prioritize limiting their time spent in the ED, they may not be receptive to interventions unrelated to the medical problem that prompted their ED visit. Despite the significant proportion of ED patients who present seeking care for low-acuity complaints, little study has been devoted to examining the baseline mental health of this population. Consequently, the receptiveness of this population to mental health screening and ED-based interventions is unknown. The goal of this study was to determine the prevalence of occult suicidal ideation and other mental health disorders among ED patients presenting with low-acuity chief complaints, along with the receptiveness of these patients to ED-based mental health interventions.

## METHODS

### Study Design, Setting, and Selection of Participants

This prospective cross-sectional survey study was performed in the Cooper University Hospital ED, an urban academic department with an annual volume of about 80,000 patients per year, which provides care for a socioeconomically diverse community in the Northeastern United States. Medical care is provided by either an attending physician or by residents or nurse practitioners under the supervision of an attending physician. The study was approved by the institutional review board, and all subjects provided signed informed consent to participate.

Patients were eligible for participation if they were aged 18 years or older and had an Emergency Severity Index (ESI) triage score of 4 or 5, indicating a low-acuity presentation, as documented by a triage nurse experienced in the use of the ESI system. Approximately 25% of the patient visits evaluated in the ED met this definition for a low-acuity presentation. Patients were excluded if they did not speak English, if they suffered from dementia or other cognitive impairment, if they presented to the ED for treatment of an acute psychiatric emergency, if they were intoxicated, or if they were incarcerated at the time of their ED visit.

Research assistants (RA) were initially trained in the study methods via a didactic lecture. An investigator then provided additional individualized training and observation until they deemed each RA proficient at independently screening subjects, obtaining informed consent, and performing data collection, after which the RAs independently performed these tasks. Subjects were enrolled between 9 a.m. and 10 p.m. during randomly selected two-hour blocks, seven days a week, between June 2015 and April 2016. We used random time-block sampling to minimize sampling bias due to convenience sampling, given limited resources available for data collection.

### Measurements

Participants completed two previously validated mental health screening instruments, the Patient Health Questionnaire for Depression and Anxiety (PHQ-4) and the Suicide Behaviors Questionnaire–Revised (SBQ-R).^10^,^11^ The PHQ-4 has been validated as a screening tool for depression and anxiety in both general and primary-care populations. Participants with scores of three or more (out of six) on either the anxiety or depression subscales were considered to be at risk for these specific disorders. Overall PHQ-4 scores of 0–2 indicate no psychological distress, 3–5 indicate mild distress, 6–8 indicate moderate distress, and scores of 9–12 indicate severe psychological distress.^10^,^12^ The SBQ-R is a tool that has been used to detect suicidality in both a general population and among patients with known, mental health disorders. A score of seven or greater was considered to identify individuals at risk of suicide.^11^ When RAs identified a patient as being at high risk of suicide, they alerted the treating ED clinician to this information, and the clinician determined what immediate steps, if any, would be taken as a result.

**Population Health Research Capsule**

What do we already know about this issue?

Symptoms of severe depression and occult suicidality are common among emergency department patients, and screening of ED patients for suicidal thoughts is required by the Joint Commission.

What was the research question?

Among low-acuity ED patients, how common are suicidal thoughts and how willing are patients to address them during the ED visit?

What was the major finding of the study?

Of 816 participants, 11% were at high risk for suicide. Many were receptive to addressing mental health issues during the ED visit.

How does this improve population health?

These findings suggest that screening low acuity ED patients for mental health concerns may be useful, though studies assessing the impact of screening on patient-oriented outcomes are needed.

Participants also provided information regarding the use and abuse of alcohol, tobacco, recreational drugs, and prescription drugs used for reasons other than prescribed. We defined binge drinking as five or more drinks in one sitting for men, and four or more for women. Additionally, patients were asked how important it was for providers to address problems related to both mental health and substance abuse during their current ED visit (for example, “How important is it that today’s emergency department visit address any mental health concerns you may have?”), and they could choose from the following responses: not important, minor importance, important, or very important.

The study instrument was assessed for content validity by a panel of individuals with expertise in urban health, barriers to healthcare access, ED care, and survey administration. One study author administered the survey during a pilot phase prior to beginning enrollment with no changes made to the survey instrument afterward.

### Statistical Analysis

We estimated that a sample size of at least 800 participants would provide a 2% margin of error based on a 95% confidence interval for the detection of occult suicidality, assuming an estimated prevalence of 8% among patients within an urban ED population.^13^ Study data were saved in a secure electronic database created using REDCap, and were analyzed in 2017.^14^ Descriptive data are presented, including proportions, median with interquartile range, and mean with standard deviation. We performed no imputation for missing data, and we excluded from analyses cases with missing data, relying on the missing data on a pair-wise basis. We used chi-square testing to compare data between categorical variables. P values < 0.05 were considered statistically significant, and we did not adjust p values for the performance of multiple comparisons. We performed data analysis using SPSS v 20.0 (IBM Corp, Armonk, NY).

## RESULTS

There were 14,571 low-acuity visits during the study period. Of these, 2,016 presented during approximately 400 two-hour enrollment windows and were screened for inclusion. From these potentially eligible patients, 328 were excluded: 195 did not speak English; 114 were intoxicated; nine were cognitively impaired; and 10 were prisoners. Of the remaining 1,688 eligible patients, 816 (48%) agreed to participate. Participants were diverse with respect to race, ethnicity, and insurance status, and 22% reported a past medical history of depression (Table 1).[Table t2-wjem-19-573]

Within this cohort, 27% of patients screened positive for anxiety, and 25% screened positive for depression, including 17% of those participants with no known history of depression. Evidence of moderate psychological distress was present in 9%, and severe psychological distress was present in another 13%. Eleven percent of all participants were found to be at significant risk of suicide (SBQ-R ≥ 7), and 5% of those with no history of depression or bipolar disorder were at risk of suicide. Race and sex were not associated with suicide risk, though risk of suicide was associated with a past history of depression (32% vs 5%, p < 0.001) or bipolar disorder (38% vs 9%, p <0.001 for both). Suicide risk was also associated with a reported history of heroin use (35% vs. 10%, p < 0.001) and cocaine use (32% vs. 10%, p = 0.001). Binge drinking monthly or more was also weakly associated with risk for suicide (15% vs 10%, p = 0.40).

Among participants at risk of suicide based on the SBQ-R, 35% felt that it was either important or very important for suicidal thoughts to be addressed during the ED visit (Table 3). Thirteen percent felt that addressing suicidal thoughts in the ED was not very important, and 52% felt that it was not important at all. Of those subjects found to be at significant risk of depression 53% felt that it was important or very important for the ED visit to address mental health concerns, while 16% thought this was not very important and 31% thought this was not important at all. Among the 105 participants with severe psychological distress, 67 (64%) felt that addressing mental health concerns in the ED was important or very important.

Reported substance abuse was common within this cohort, with 23% of participants binge drinking alcohol monthly or more. About one-third (280, 34.5%) used tobacco products daily or almost daily. Twelve percent reported using recreational drugs monthly or more, including 6% who used these drugs daily or almost daily. Six percent reported abusing prescription drugs for reasons other than prescribed. Of the 179 participants with monthly binge alcohol drinking, just 31 (17%) thought that receiving assistance with substance abuse during the ED visit was important or very important. Of the 96 participants with monthly recreational drug use, 33 (34%) felt that addressing this issue in the ED was important or very important.

## DISCUSSION

In this large cohort of ED patients with low-acuity chief complaints, we observed that a significant proportion of patients who presented with a chief complaint not suggestive of psychiatric disease had mental health concerns that were apparent on screening, including 11% with a significant risk of suicide. Between approximately one-third and two-thirds of these patients with evidence of significant psychiatric concerns were open to ED-based interventions targeting their mental health.

These results are consistent with prior studies describing the under-diagnosis of depression and other mental health disorders among ED patients,^15^, ^16^ along with the presence of passive suicidal ideation among approximately 6%–12% of patients who are evaluated in the ED for non-psychiatric reasons.^5^,^6^,^13^,^17^ The effectiveness of ED-based screening programs can be improved through the use of performance improvement methodologies, as well as careful training of the staff members who perform these screenings.^17^ These methods are relatively resource-intensive, however, thus raising the question of whether screening every ED patient for suicidality is necessary.

In particular, patients with low-acuity complaints are often managed through ED triage and evaluation processes that are distinct from higher-acuity patients, resulting in their rapid evaluation and discharge that limits opportunities for careful mental health screening. Our results suggest that even among these lower-acuity patients, mental health screening may have value. However, perhaps due to the expectation that visits for low-acuity complaints would be both rapid and focused, a significant proportion of patients at risk for suicide, based on their SBQ-R score, indicated that they did not want to address mental health concerns during the ED visit. Significant additional work is needed to ensure that the benefits of screening justify the costs involved.

Before any widespread screening program is initiated, it is important to both confirm the existence of effective treatments such that the anticipated benefits of screening will outweigh anticipated harms, and to assess the resources required to achieve these outcomes. With respect to ED-based screening for suicidality there are a number of existing interventions that may be helpful in reducing the incidence of suicide among at-risk patients. These include linkage to specialist care, the development of a safety plan, and counseling about modifiable risk factors such as access to firearms.^18^,^19^

However, very few studies have evaluated the long-term impact of these interventions on patient outcomes. For example, despite showing the feasibility of using a mobile crisis team to establish linkage to care among suicidal patients who are discharged from the ED, this intervention did not improve long-term symptom burdens.^20^ Another recent study of patients with active or recent suicidal ideation showed that combining universal suicide-risk screening with interventions aimed at reducing suicidal behavior was associated with a decrease in suicide attempts at one year, suggesting that coupling screening with a defined intervention is more effective than screening alone.^21^ Future studies are needed to explicitely assess the impact of mental health screening programs on patient-oriented health outcomes, such as suicide completion and symptom burden.

Additionally, we did not assess the resource burden required to effectively screen a non-targeted population of ED patients for mental health disorders, though this is another key question that ideally would have been addressed before widespread screening was implemented.^7^ Relevant ED costs include staff time for screening, opportunity costs of screening during brief ED visits, time required to follow up on patients who screen positive, and the potential for increasing the length of stay when patients screen positive. Other relevant costs include the expense and limited availability of mental health resources that get devoted to those who screen positive, risks of false-positive screening, and potential harms to patients who are labeled as having a mental health diagnosis. These concerns are all particularly important when screening an untargeted population in which the disorder of interest is relatively uncommon, and when the effectiveness of subsequent interventions is not well established.

## LIMITATIONS

This study is subject to several important limitations that should be considered when interpreting these results. First, participants were recruited from a single academic ED within the U.S. between 9 a.m. and 10 p.m. While most low-acuity patients present to EDs during daytime hours and our sample was diverse with respect to race, ethnicity, and access to healthcare, results from other populations may differ.^22^ In particular, it is likely that patients presenting during nighttime hours with low-acuity complaints systematically differ from patients presenting during daytime and evening hours. Second, about half of the patients we approached for enrollment declined, possibly reflecting a combination of patient discomfort due to pain or other acute symptoms, as well as a limited tolerance for anything that might have increased the ED visit duration among low-acuity patients expecting rapid evaluation and discharge.

Therefore, it is likely that sampling bias impacted our observed results. For example, rates of occult mental health disorders may differ between respondents and non-respondents. Furthermore, even if rates of occult mental health disorders are similar between respondents and non-respondents, there may be differences between these groups in patients’ willingness to report mental health symptoms. Also, compared to those patients who chose not to participate, study participants might have been more open to receiving ED-based interventions for mental health disorders not related to their chief complaint, which would have limited the utility of routine mental health screening among this population.

Additionally, we used the ESI triage system as applied by an experienced triage nurse to identify a low-acuity patient cohort. The admission rate of study participants was just 7%, which supports this assumption, though ESI is not perfect in this regard. We used the SBQ-R to assess suicidality, which has been validated to predict long-term suicidal behavior. While this instrument is one of several recommended by the Joint Commission for use as a screening tool, it has not been validated for use in predicting short-term suicide completion.^7^ Similarly, our questions addressing the perceived importance of addressing mental health concerns have not been previously validated. Finally, while we determined the prevalence of occult mental health disorders among this patient cohort and assessed receptiveness to ED-based interventions aimed at addressing these issues, we did not explore either the effectiveness or the risks of any specific interventions. Both steps should be undertaken prior to implementing any intervention aimed at addressing these issues.

## CONCLUSION

Among this cohort of ED patients presenting for non-psychiatric reasons with low-acuity chief complaints, a significant portion were at risk for suicidal behavior. Further, approximately one-quarter of patients screened positive for moderate to severe psychological distress. A substantial proportion of patients with mental health disorders unrelated to their chief complaints were open to addressing these disorders during the ED visit.

## Figures and Tables

**Table 1:** Characteristics of patients (N=816) with low-acuity presentation to the emergency department who participated in mental health screening.

Patient characteristics	Frequency; N (%)
Sex	
Female	466 (57)
Age, median (IQR)	34 (26–49)
Race	
Black	397(49)
White	216 (27)
Other	181 (22)
Ethnicity	
Hispanic	220 (27)
Insurance status	
Private insurance	80 (10)
Medicare	98 (12)
Medicaid	442 (54)
Uninsured	107 (13)
Other/no answer	89 (11)
Has a primary care provider	588 (72)
Mental health history	
Depression	181 (22)
Bipolar disorder	66 (8)
Schizophrenia	19 (2)

*IQR*, interquartile range.

**Table 2 t2-wjem-19-573:** Results of mental health screening using the SBQ-R for suicidality and PHQ-4 for psychological distress.

Survey Metric	N (%)
Suicidality (n = 802)	
SBQ-R Total score ≥7	89 (10.9)
Psychological Distress (n = 772)	
PHQ-4 Score 3–5 (Mild)	123 (15.2)
PHQ-4 Score 6–8 (Moderate)	74 (9.1)
PHQ-4 Score 9–12 (Severe)	105 (12.9)
PHQ-4 Anxiety Score ≥3	209 (25.6)
PHQ-4 Depression Score ≥3	196 (24.1)

*SBQ-R,* Suicide Behaviors Questionnaire-Revised; *PHQ-4,* Patient Health Questionnaire-4.

**Table 3:** Perceived importance of addressing psychiatric concerns among patients reporting mental health symptoms.

Mental health screening result	Not important at all	Not very important	Important	Very important
How important is it that today’s Emergency Department visit address any suicidal thoughts or ideations you may have? N (%)				
Suicidal Risk (SBQ-R ≥7); n = 89	46 (52)	12 (13)	10 (11)	21 (24)
How important is it that today’s Emergency Department visit address any mental health concerns you may have? N (%)				
Psychological Distress				
PHQ-4 Score 3–5 (Mild) ; n = 122^1^	53 (43)	16 (13)	24 (20)	29 (24)
PHQ-4 Score 6–8 (Moderate); n = 74	34 (46)	11 (15)	14 (19)	15 (20)
PHQ-4 Score 9–12 (Severe); n = 105	23 (22)	15 (14)	21 (20)	46 (44)
PHQ-4 Anxiety Score ≥3; n = 209	72 (34)	28 (13)	40 (19)	69 (33)
PHQ-4 Depression Score ≥3; n = 195^1^	60 (31)	31 (16)	40 (21)	64 (33)

*SBQ-R,* Suicide Behaviors Questionnaire-Revised; *PHQ-4,* Patient Health Questionnaire-4.

1 One case excluded due to missing data.
